# P-2171. Comparing Saliva and Nasal Mid-turbinate Swabs for Detection of Respiratory Syncytial Virus (RSV), February 1–April 15, 2024, New Vaccine Surveillance Network

**DOI:** 10.1093/ofid/ofae631.2325

**Published:** 2025-01-29

**Authors:** Anjana Sasidharan, Montserrat Santos, Minati Dhar, Bri Maldonado, Benjamin R Clopper, Heidi L Moline, Sydnie Petty, Dithi Banerjee, Rangaraj Selvarangan

**Affiliations:** Childrens Mercy Hospital, Missouri, Kansas; Children's Mercy Research Hospital, Kansas City, Missouri; Children's Mercy Hospital, Kansas City, Missouri; Children's Mercy Hospital, Kansas City, Kansas City, Missouri; US Centers for Disease Control & Prevention, Buffalo, New York; Centers for Disease Control and Prevention, Atlanta, Georgia; MCC-Longview, Raytown, Missouri; Children's Mercy Hospital, Kansas City, Missouri; Children’s Mercy Kansas City, Kansas City, Missouri

## Abstract

**Background:**

Current diagnostic methods for RSV and other respiratory viruses primarily rely on reverse transcription polymerase chain reaction (RT-PCR) testing of nasopharyngeal (NPS) or nasal mid-turbinate (MTS) swabs. Recent data in adults suggest saliva increases diagnostic yield of RSV. However, the potential of saliva as a specimen type for detecting RSV in children remains understudied. We compared the diagnostic yield of saliva and paired MTS samples for RSV detection in children.

**Methods:**

Between February 1 to April 15, 2024, a total of 206 paired MTS and saliva samples obtained using the SalivaBio Children’s swab were collected on the same day from subjects under the age of 18 years presenting with acute respiratory illness symptoms. All MTS samples underwent testing using the QIAstat-Dx Respiratory SARS-CoV-2 Panel, which identifies RSV A+B cases. Subsequently, saliva samples from RSV-positive cases identified by MTS testing were extracted using the Kingfisher Apex platform. Positive RSV cases were subtyped by RT-PCR.

**Results:**

Among 206 paired MTS and saliva specimens tested, RSV A/B was detected in 8 MTS and 7 saliva specimens. Saliva detected RSV A/B in 7 out of 8 (87.5%) samples that tested positive by MTS swabs. Both MTS and saliva tested negative for RSV in 197/206 specimens tested. The median (IQR) Ct values for paired MTS swabs and saliva samples were 25.05 (21.82 – 32.72) and 31.9 (27.45 – 34.48), respectively. The sample missed by saliva had a high Ct value of 36.6. Saliva detected an additional RSV-A positive case with a Ct value of 33.03, which was negative by the MTS swab. Overall concordance of saliva with MTS was 99% (204/206).

**Conclusion:**

Although the number of RSV detections was low, preliminary findings from this analysis suggest high concordance between MTS and saliva for detection of RSV but that the addition of saliva does not increase diagnostic yield in children.

**Disclosures:**

Rangaraj Selvarangan, BVSc, PhD, D(ABMM), FIDSA, FAAM, Abbott: Grant/Research Support|Abbott: Honoraria|BioMerieux: Grant/Research Support|Cepheid: Grant/Research Support|Diasorin: Grant/Research Support|GSK: Advisor/Consultant|Hologic: Grant/Research Support|Luminex: Grant/Research Support|Qiagen: Grant/Research Support

